# Identification of potential biomarkers for predicting the early onset of diabetic cardiomyopathy in a mouse model

**DOI:** 10.1038/s41598-020-69254-x

**Published:** 2020-07-23

**Authors:** Rabia Johnson, Xolisa Nxele, Martin Cour, Nonhlakanipho Sangweni, Tracey Jooste, Nkanyiso Hadebe, Ebrahim Samodien, Mongi Benjeddou, Mikateko Mazino, Johan Louw, Sandrine Lecour

**Affiliations:** 1 Biomedical Research and Innovation Platform (BRIP), South African Medical Research Council (SAMRC), Tygerberg, 7505 South Africa; 2 Division of Medical Physiology, Faculty of Health Sciences, Stellenbosch University, Tygerberg, South Africa; 3 Department of Biotechnology, University of Western Cape, Cape Town, South Africa; 4 Hospices Civils de Lyon, Hôpital Edouard Herriot, Service de Médecine Intensive-Réanimation, Lyon Cedex 03, France; 5 Hatter Institute for Cardiovascular Research in Africa (HICRA), Faculty of Health Sciences, University of Cape Town, Observatory, South Africa; 6 Department of Anaesthesia, Faculty of Health Sciences, University of Cape Town, Observatory, South Africa; 7 Biostatistics Research Unit, South African Medical Research Council (SAMRC), Cape Town, South Africa; 8 Department of Biochemistry and Microbiology, University of Zululand, KwaDlangezwa 3886, Richards Bay, South Africa

**Keywords:** Molecular biology, Biomarkers, Cardiology, Medical research, Risk factors

## Abstract

Type 2 diabetes (T2D) is characterized by metabolic derangements that cause a shift in substrate preference, inducing cardiac interstitial fibrosis. Interstitial fibrosis plays a key role in aggravating left ventricular diastolic dysfunction (LVDD), which has previously been associated with the asymptomatic onset of heart failure. The latter is responsible for 80% of deaths among diabetic patients and has been termed diabetic cardiomyopathy (DCM). Through in silico prediction and subsequent detection in a leptin receptor-deficient db/db mice model (db/db), we confirmed the presence of previously identified potential biomarkers to detect the early onset of DCM. Differential expression of Lysyl Oxidase Like 2 (*LOXL2*) and Electron Transfer Flavoprotein Beta Subunit (*ETFβ*), in both serum and heart tissue of 6–16-week-old db/db mice, correlated with a reduced left-ventricular diastolic dysfunction as assessed by high-resolution Doppler echocardiography. Principal component analysis of the combined biomarkers, *LOXL2* and *ETFβ*, further displayed a significant difference between wild type and db/db mice from as early as 9 weeks of age. Knockdown in H9c2 cells, utilising siRNA of either LOXL2 or ETFβ, revealed a decrease in the expression of Collagen Type I Alpha1 (*COL1A1*), a marker known to contribute to enhanced myocardial fibrosis. Additionally, receiver-operating curve (ROC) analysis of the proposed diagnostic profile showed that the combination of *LOXL2* and *ETFβ* resulted in an area under the curve (AUC) of 0.813, with a cut-off point of 0.824, thus suggesting the favorable positive predictive power of the model and further supporting the use of LOXL2 and *ETFβ* as possible early predictive DCM biomarkers.

## Introduction

Diabetes affects 463 million people worldwide and this number is said to increase to 700 million by 2045^1^. Diabetes and its complications are inextricably linked to cardiovascular dysfunction, which is currently the leading cause of mortality worldwide, affecting 17.9 million individuals^2^. This association was first reported in the Framingham Heart study and since then numerous reports have come to the forefront, confirming a 2–4 times increased susceptibility of diabetic individuals to heart failure (HF)^3,4^. Coronary artery disease (CAD) is the major type of CVD responsible for HF in diabetic individuals, however diabetic cardiomyopathy (DCM) is an established complication of diabetes mellitus (DM) existing in the absence of CAD or hypertension^5^. Furthermore, DCM is referred to as the silent killer, due to the manifestation of a long subclinical period in which the disease exists with no overt clinical symptoms^6–8^.

DCM proceeds in three different stages: asymptomatic, diastolic and systolic stages, respectively. In its asymptomatic stage, the disease is characterized by abnormal left ventricular relaxation, referred to as left ventricular dysfunction (LVD). These functional abnormalities can be seen in 40–60% of asymptomatic diabetic cases using Doppler echocardiography, Tissue Doppler Imaging (TDI) echocardiography analysis or Speckle Track Imaging (STI) echocardiography^9^. Also, diabetic patients with subclinical diastolic dysfunction (DD) have a 5-year motility rate of 30% compared to the 12.1% for non-diabetic patients with no diastolic dysfunction^10^. As DCM enters its later stage, it progresses from DD to overt heart failure with preserved ejection fraction, which has no proven effective treatment nor a biomarker available that can detect the early onset of DCM^8^. TDI- and STI echocardiograph analysis are the only imaging technologies available to detect DCM in its asymptomatic stage. However, these techniques are not always available, especially in developing countries. Current diagnostic markers include N-terminal pro b-type brain natriuretic peptide (NT-proBNP) and cardiac troponin T (cTn-T). However, none of these markers are able to detect DCM, either in its asymptomatic stage or early enough to have a therapeutic impact.

As such, there is a need for a prognostic marker that can improve early detection of DCM prior to the onset of irreversible complications. However, despite numerous clinical and pharmacological intervention studies, DCM remains elusive warranting new strategies to identify asymptomatic detection of the disease state. In this study, using an in silico predictive method the combination use of two possible biomarkers was identified, Lysyl Oxidase Like 2 (LOXL2) and Electron Transfer Flavoprotein Beta Subunit (ETFβ). The presence of the identified markers was then screened in the serum of db/db mice with confirmed LVDD.

## Results

### DCM biomarker prediction

**Figure 1:**
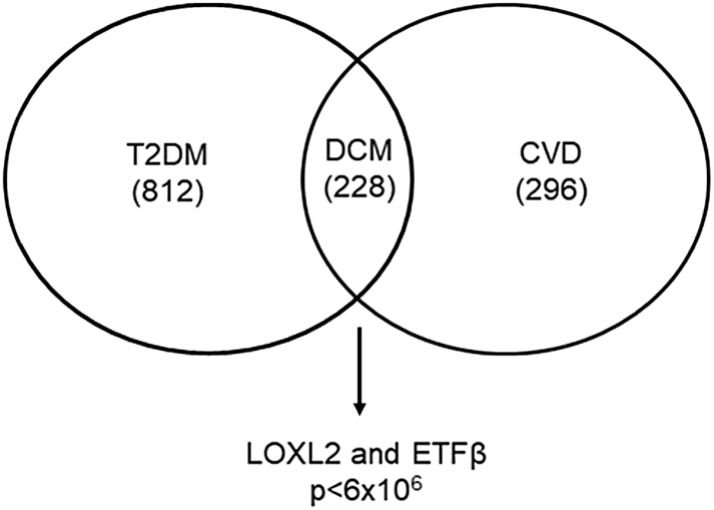
Venn diagram of differentially expressed genes (DEGs). Within the type 2 diabetes mellitus (T2DM) and cardiovascular disease (CVD) datasets, 812 and 296 candidate genes were identified respectively. Integration of the T2DM and CVD datasets, a diabetic cardiomyopathy (DCM) dataset was generated, in which 2 possible candidate genes were identified based on their Wilcoxon score (*p* value  < 6 × 10^–6^).

Meta-analysis of publicly available microarray data sets using ArrayExpress resulted in approximately 15,485 and 16,450 common Ensemble IDs for T2DM and CVD, respectively. Upon statistical differential expression analysis, using the QD binning approach, 812 and 296 differential expressed genes (DEGS) were identified for T2DM and CVD, respectively. Thereafter, using a similar quantile discretization (QD) analysis, T2DM and CVD genes were integrated, and 228 common genes were identified. Due to limited serum availability in the animal study and to prevent bias, the top 2 genes were selected according to their Wilcoxon score (*p* value < 6 × 10^–6^), namely *LOXL2* and *ETFβ*, for further downstream analysis (Fig. 1).

### Morphometric analysis of risk factors associated with DCM

To assess the presence of cardiac risk factors in our db/db model, fasting blood glucose levels (FBG) and serum lipids levels were investigated (Table 1). In this study, diabetic (db/db) mice presented with significantly increased body weight/tibia length at age 6, 9, 11 and 16 weeks when compared to the non-diabetic (db/ +) control. There were no differences in heart weight/tibia length (data not shown), as well as the heart rate between db/db mice and aged-matched controls (*p* > 0.05). Fasting blood glucose levels, cholesterol, triglycerides and low-density lipoproteins (LDL) of db/db mice were significantly elevated from 9 weeks onwards when compared to their age-matched controls. The observed effects are characteristic of metabolic syndrome that continued throughout the study.

**Table 1:** Risk markers of cardiovascular dysfunction in db/db mice compared to their wild type control (db/ +).

	Wild type (db/ +)					Obese (db/db)				
	6 weeks	9 weeks	11 weeks	14 weeks	16 weeks	6 weeks	9 weeks	11 weeks	14 weeks	16 weeks
BW (g)/tibia length (mm)	1.21 ± 0.03	1.28 ± 0.06	1.34 ± 0.03	1.38 ± 0.02	1.33 ± 0.03	1.68 ± 0.06***	1.96 ± 0.03***	2.11 ± 0.07***	1.80 ± 0.14***	1.69 ± 0.11**
FBG (mmol/L)	8.33 ± 0.58	7.09 ± 0.29	8.91 ± 5.15	6.94 ± 0.71	10.46 ± 8.07	12.50 ± 0.52	22.74 ± 2.76***	28.30 ± 1.88***	29.50 ± 1.44***	27.33 ± 9.20***
Cholesterol (mol/L)	1.90 ± 0.07	2.37 ± 0.41	1.75 ± 0.03	1.85 ± 0.05	2.12 ± 0.17	3.90 ± 0.21***	3.45 ± 0.59*	3.79 ± 0.24**	3.90 ± 0.34**	3.77 ± 0.20***
LDL (mmol/L)	0.30 ± 0.00	0.30 ± 0.00	n/a	0.30 ± 0.00	0.30 ± 0.00	0.55 ± 0.05**	0.37 ± 0.08	n/a	0.50 ± 0.15	0.53 ± 0.10**
Triglycerides (mmol/L)	0.84 ± 0.14	0.77 ± 0.08	n/a	1.10 ± 0.13	0.78 ± 0.17	2.43 ± 0.22**	2.10 ± 0.56	n/a	3.11 ± 0.36	1.86 ± 0.65**

*BW* body weight, *LDL* low-density lipoprotein, *FBG* fasting blood glucose levels.

*p ≤ 0.015, **p < 0.01 ***p ≤ 0.001 versus wild type control (db/ +) at the same time point.

### mRNA expression analysis of LOXL2 and ETFβ in cardiac tissue

**Figure 2:**
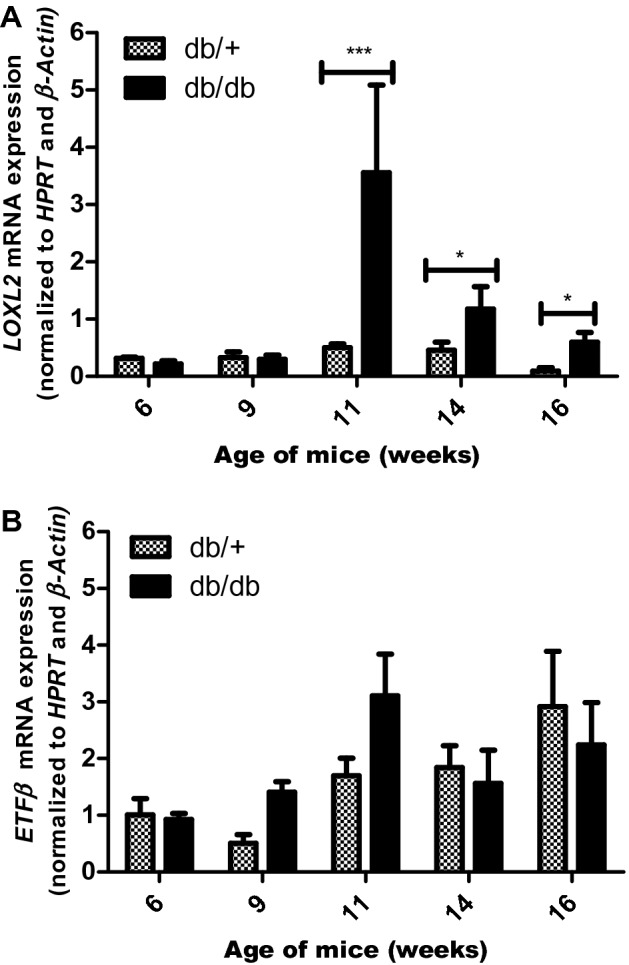
mRNA expression of LOXL2 and ETFβ in hearts tissue of 6–16-week-old db/db mice. Differential mRNA expression of (**A**) Lysyl Oxidase Like 2 (*LOXL2*) and (**B**) Electron transfer flavoprotein subunit beta (*ETFβ*) in heart tissue of leptin receptor-deficient mice (db/db) compared to aged matched lean control (db/ +). Results are expressed as mean ± SEM of n = 4–8 animals per group. *p < 0.05 and ***p ≤ 0.001 compared to wild type control (db/ +).

The expression of the two candidate biomarkers was verified in cardiac tissue of leptin-receptor-deficient mice. The results obtained showed a marked increase in *LOXL2* mRNA expression at 11-weeks (sevenfold, *p* < 0.001) when comparing db/db mice to the db/+ control. This expression difference was maintained at both 14-weeks (fourfold, *p* < 0.1) and 16-weeks (sixfold, *p* < 0.01) (Fig. 2A). *ETFβ* mRNA expression, on the other hand, tends to increase until 11-weeks (1.8-fold) when comparing db/db mice to db/+ control, after which a tendency to decrease in expression was observed at 14- (1.2-fold) and 16-weeks (1.3-fold), (Fig. 2B).

### LOXL2, ETFβ and proBNP protein are expressed in the serum of obese mice

**Figure 3:**
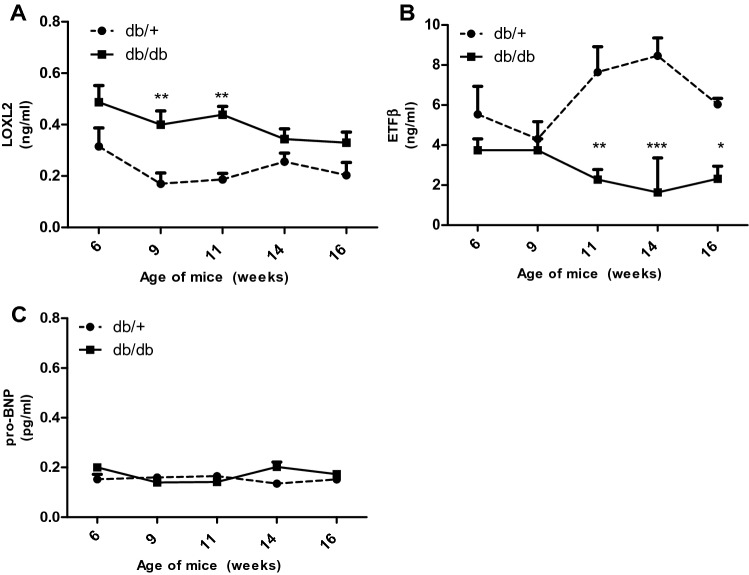
Expression of serum LOXL2 and ETFβ in 6–16-week-old Leptin receptor-deficient db/db mice and aged matched controls. (**A**) Lysyl Oxidase Like 2 (LOXL2), (**B**) Electron transfer flavoprotein subunit beta (ETFβ) and (**C**) N-terminal pro b-type natriuretic peptide protein expression in serum of leptin receptor-deficient mice (db/db) compared to aged matched lean control (db/ +). Results are expressed as mean ± SEM of n = 8 animals per group. *p < 0.05, **p < 0.01 and ***p < 0.001 compared to wild type control (db/ +) at the same point.

To explore the effect of LOXL2 and ETFβ as a prognostic marker, we tested if the identified proteins are released from the cardiac interstitial space into the circulation. Results obtained showed that LOXL2 levels were significantly elevated in the serum of obese animals at 9- (*p* < 0.01), and 11-weeks (*p* < 0.01) when compared to age-matched controls, whereas serum levels at 6-, 14- and 16-weeks showed an increasing trend (*p* > 0.05) (Fig. 3A). Next serum protein levels of ETFβ were investigated and data obtained showed that protein levels of db/db mice showed no change at 6 and 9 weeks (*p* > 0.05) and were significantly reduced at 11- (*p* < 0.01), 14- (*p* < 0.001) and 16-weeks (*p* < 0.05) when compared to the control (Fig. 3B). Interestingly, measurements of serum NT-pro-BNP (the known HF biomarker) was unchanged between 6 and 16 weeks. (*p* > 0.05) (Fig. 3C).

### High resolution Doppler echocardiography analysis as the gold standard to detect diabetic cardiomyopathy

**Figure 4:**
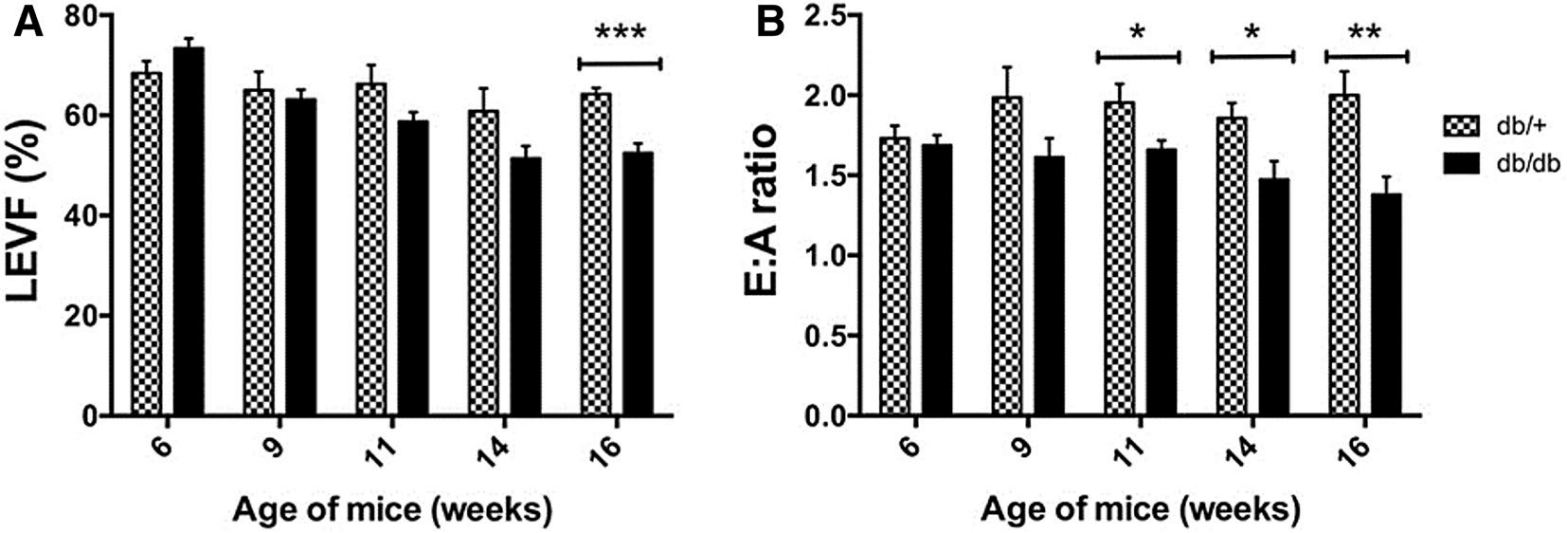
High-resolution Doppler echocardiography analysis in 6–16-week-old Leptin receptor-deficient db/db mice and their age matched db/+ heterozygous non-diabetic control group. (**A**) Measurement of left ventricular ejection fraction (LVEF) as a marker of systolic function; (**B**) Measurement of mitral E:A ratio as a marker of left ventricular diastolic function. Results are expressed as mean ± SEM of 6–8 animals per group. Statistical significance was evaluated using Mann–Whitney test . *p < 0.05, **p < 0.01, and ***p < 0.001 compared to aged matched wild type control.

High-resolution Doppler echocardiography analysis was used to record structural and functional measurements of left ventricular remodeling in db/db and control mice as summarized in Fig. 4 and Supplementary Table S1. No significant difference in left ventricular internal diameter, left ventricular posterior wall thickness-end diastole and left ventricular mass at 6, 9, 11,14 and 16 weeks of age were observed in db/db compared to db/+ control (*p* > 0.05). As shown in Fig. 4, significant impairement in LV systolic dysfunction was observed at 16 weeks in db/db mice while diastolic dysfunction, as assessed by a decrease in mitral E:A ratio, was diagnosed as early as 11 weeks (Fig. 4). Diastolic dysfunction was also confirmed by a significant increase in E wave deceleration time (E_DT_) and in isovolumic relaxation time (IVRT) in db/db compared to db/+ at both 14 and 16 weeks (Supplementary Table S1).

### Serum LOXL2, ETFβ and NT-proBNP correlation analysis with mitral E:A ratio

**Figure 5:**
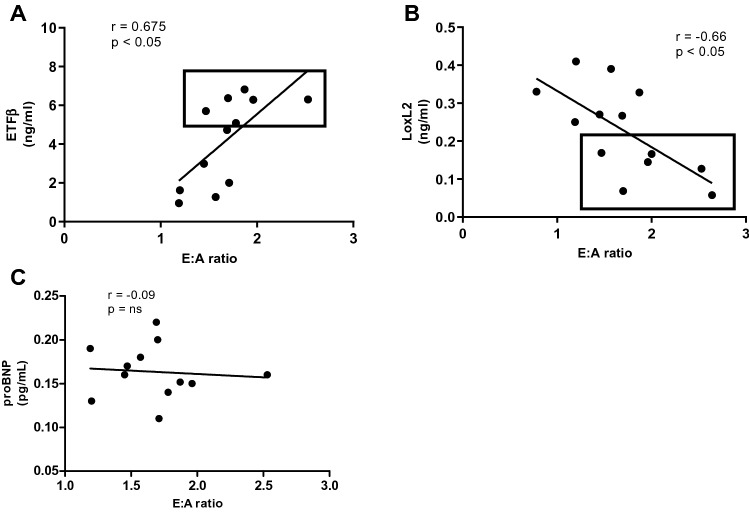
Correlation analysis of serum LOXL2, ETFβ and proBNP with mitral E:A ratio in Leptin receptor-deficient db/db mice at 16 weeks. Mitral E:A ratio is a marker of left ventricular diastolic dysfunction, (**A**) Lysyl Oxidase Like 2 (LOXL2), was negatively and (**B**) Electron transfer flavoprotein subunit beta (ETFβ) was positively correlated with mitral E:A ratio, while no correlation was observed with (**C**) N-terminal pro b-type natriuretic peptide (proBNP).

In clinical practice the mitral E:A ratio is currently used as an indication of the degree of HF, guiding the implementation of interventions. Therefore, we examined the correlation between serum levels of the two candidate biomarkers (LOXL2, ETFβ) as well as NT-proBNP, using the Pearson’s correlation test, and observed no linear relationship between serum levels of NT-proBNP and the mitral E:A ratio (r = 0.05) (Fig. 5C). However, a strong (downhill) negative linear relationship was observed between serum LOXL2 levels and the E: A ratio (r = − 0.65, *p* = 0.01), while a positive correlation was observed between ETFβ and the E: A ratio (Pearson correlation, r = 0.65, *p* < 0.001) (Fig. 5A,B). Based on the correlation analysis, we suggest that LOXL2 and ETFβ could serve as potential biomarkers for the diagnosis of DCM.

### siRNA analysis of ***LOXL2*** and ***ETF***${\varvec{\beta}}$

**Figure 6:**
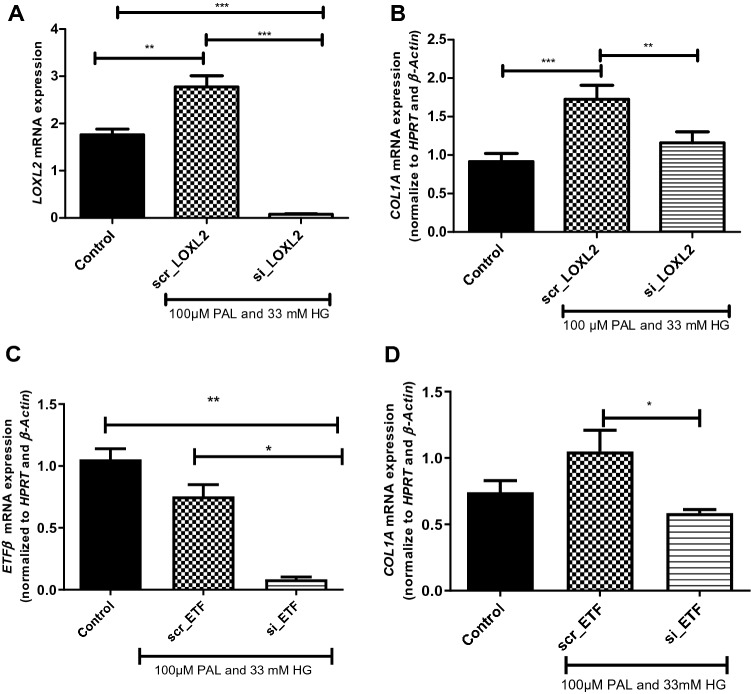
Effect of LOXL2 knockdown on *Col1A* and *ETFβ* expression. The degree of knockdown after H9c2 cardiomyocytes transfected with (**A**) si*LOXL2* and (**C**) s*iETFβ* was exposed to 100 µM Palmitate (PAL) and 33 mM glucose (HG). The relative mRNA expression of the fibrosis gene, *COL1A* in (**B**) *LOXL2* and (**D**) *ETFβ* after transfection of cells with either small interfering RNA (siRNA) or scrambled RNA (scrRNA). The expression levels were normalized relative to the control. Statistical significance was evaluated using the one-way ANOVA with a Tukey Post-hoc t-test. This graph represents the SEM of triplicate samples. *p* < 0.05, *p* < 0.01 and *p* < 0.001 when compared to control or scrambled control.

In order to assess the functional role *LOXL2* and *ETFβ* may play in collagen crosslinking, H9c2 cells treated with siRNA were exposed to high glucose (HG) and palmitate (PAL). Results obtained showed that HG and PAL significantly increased scr_*LOXL2*, while decreasing scr_*ETFβ* when compared to the untreated control (*p* < 0.001) (Fig. 6A,C). Alternatively, cells treated with either si_*LOXL2* or si_*ETFβ* showed significantly decreased mRNA expression when compared to the control (*p* < 0.001 and *p* < 0.01, respectively) (Fig. 6A,C). Subsequently, the role of *LOXL2* and *ETFβ* on the expression on *COL1A* was assessed. Results obtained showed that H9c2 cells transfected with the si_*LOXL2*, and post-treated with HG and PAL, resulted in a significant decrease in *COL1A* mRNA expression (*p* < 0.01) when compared to the scrambled (Fig. 6B). Similarly, H9c2 cells transfected with si*_ETFβ* showed significantly decreased *COL1A* mRNA expression (Fig. 6D).

### Determination of sensitivity and specificity of LOXL2 and ETFβ as a predictive biomarker

**Figure 7:**
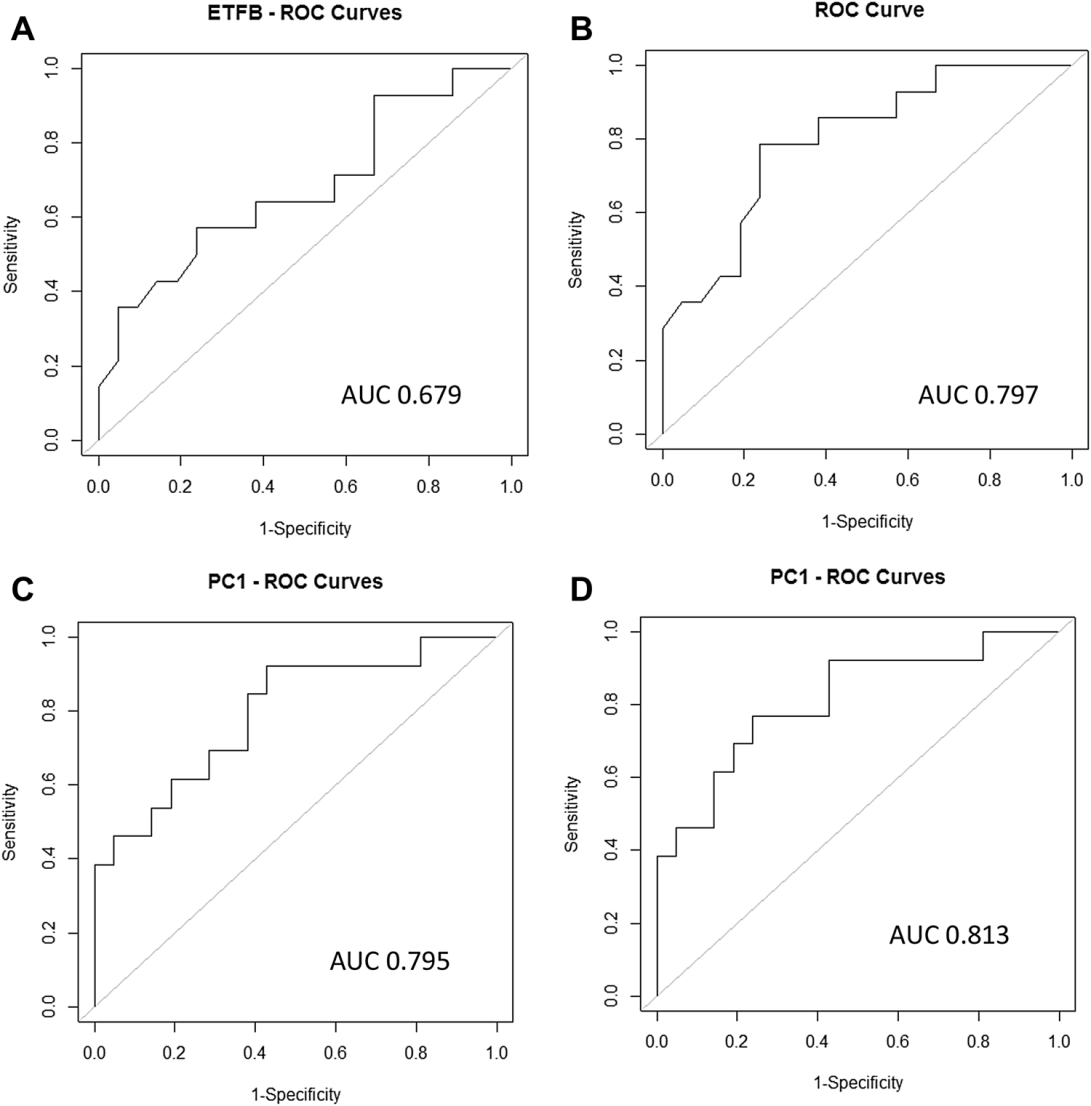
Receiver operating characteristics (ROC) of LOXL2 and ETFβ between 9 and 14 weeks. ROC curve analysis of ETFβ with (**A**) groups not specified and (**B**) groups specified. (**C**) Linear combination of LOXL2 and ETFβ when groups are not specified and when (**D**) groups are specified a good predictive cut-off point of 0.824 was obtained. Logistic regression was used to perform ROC, *p* < 0.05.

In order to evaluate the diagnostic ability of the two candidate biomarkers (LOXL2 and ETFβ) and it’s possible use in a clinical setting, a generalised linear model was used to test for significant differences (*p* < 0.001) between db/+ and db/db mice at each week and compared to that of NT-proBNP. The results obtained showed no significant differences for NT-proBNP and LOXL2 at all the evaluated time points. Conversely, ETFβ showed significant differences at week 11 and 14 (*p* < 0.01) (Table 2). Subsequently, the results for the three biomarkers (NT-proBNP, LOXL2 and ETFβ) were combined to perform principal component analysis (PCA). Results showed that the first principal component accounted for almost 89% of the variation in the data, which was the linear combination of the three biomarkers (data not shown). To conduct ROC curve analysis the mice groups were either specified (db/+ or db/db) or unspecified. Receiver Operating Characteristic (ROC) curves, for NT-proBNP and *LOXL2* generated area under the curve (AUC) values that remained non-significant in both specified and unspecified groups (data not shown). However, results obtained for ETFβ demonstrated an AUC of 0.679 when the groups were not specified (Fig. 7A), which increased to 0.797 when they were specified (Fig. 7B). These results suggested that ETFβ may be sensitive and specific enough to detect DCM.

**Table 2:** Statistical analysis for all biomarkers.

	Mice age (weeks)	t value	*p* value
NT-proBNP	6	− 2.05	0.096
	9	1.33	0.23
	11	1.85	0.1
	14	− 1.05	0.34
	16	− 1.22	0.25
ETFβ	6	1.94	0.09
	9	0.59	0.57
	11	3.91	0.001*
	14	3.67	0.001*
	16	3.08	0.022
LOXL2	6	0.11	0.92
	9	− 2.39	0.04
	11	− 4.29	0.09
	14	0.23	0.82
	16	− 1.98	0.08

*ETFβ, at 11 and 14 weeks have a significant difference (*p* = 0.01).

### Combination use of LOXL2 and ETFβ

Since there was a significant difference in the expression of both biomarkers, LOXL2 and ETFβ, between mice at 9 weeks to 14 weeks, a principal component analysis was performed by combining the two datasets to further improve their diagnostic ability. This linear combination was also used to generate ROC curves. Data presented for the linear combination of LOXL2 and ETFβ showed an AUC of 0.795 with a cutoff point of 0.789 when the groups were not specified (Fig. 7C). However, the AUC was significantly increased to 0.813 when the groups were specified, with a cutoff value of 0.824 (Fig. 7D). The latter demonstrated a good predictive power of the model, thus implying that, compared with ETFβ alone, the combination of the *ETFβ* and *LOXL2* as a biomarker is more sensitive and potentially has a higher diagnostic value.

## Discussion

Currently, there is no single prognostic test that is sensitive enough to detect the pre-clinical onset of LVDD^11,12^. Although brain natriuretic peptide (BNP) and NT-pro-BNP are well-established serum markers used to detect cardiac abnormalities, they are unable to predict the early onset of DCM^12,13^. This was confirmed in the current study. Furthermore, TDI echocardiography, Phase-Magnetic Resonance Imaging and 2D/3D-Speckle Tracking echocardiography are all non-invasive diagnostic methods with the ability to diagnose and stratify LVDD in diabetic patients. However, these tests are expensive and not done routinely as they are specialized equipment that requires trained personal which is not available in all healthcare facilities. Recently, sodium-glucose linked transporter (SGLT2) inhibitors have been recognized as a new class of drugs that can protect the diabetic myocardium. However, clinicians are currently faced with the dilemma of when to start treating diabetic individuals with SGLT2 inhibitors. Thus, a routine biomarker would greatly aid the confirmation of the presence of cardiac dysfunction and would allow an opportunity for the implementation of corrective therapy.

In the current study, we confirmed the occurrence of two potential serum biomarkers, LOXL2 and ETFβ, to detect early onset of DCM. The presence of serum LOXL2 was first reported by Yang followed by Zhao who argued that augmented LOXL2 enhances collagen crosslinking that consequently increases cardiac fibrosis^14,15^. Pertaining to ETFβ, Lu reported that decreased ETFβ expression was implicated in mitochondrial shift in substrate preference^16,17^. Also, in a review conducted by Lorenzo and colleagues, the mentioned disease pathophysiology came under investigation and the authors concluded that both processes (augmented fibrosis and mitochondrial dysfunction) play a major role in the development of diabetes-induced HF^11,18^. However, none of the mentioned studies aimed to investigate the combined use of the identified serum biomarkers. As such, our current study is the first to investigate the use of LOXL2 and ETFβ as a combination biomarker for early detection of DCM.

The db/db mouse model is a well-established heart failure animal model in which mice develop diabetes by 8 weeks of age^19,20^ with increased LDL, cholesterol and triglycerides observed by week 9^21,22^. We observed that by 6 weeks, diabetic mice weighed significantly more and presented with increased FBG, triglycerides, LDL and cholesterol levels. The latter are known features of DCM and were confirmed in a study conducted by Dludla, where the authors showed that increased FBG and lipid profiles are key risk predictors of cardiac fibrosis and subsequent HF^22^. Furthermore, it has been argued by Fang that myocardial functional changes do not progress in parallel with histological changes^8^. Instead, diabetic individuals have a long latent phase in which functional abnormalities, induced by the hyperglycaemic state, precede the onset of any structural changes. This was confirmed by Liu who argued that DCM has a long latent phase in which subtle structural modifications occur in the absence of symptomatic onset of the disease^23^. The current study confirmed this with echocardiography changes confirming the presence of reduced ejection fraction whilst subtle changes in fibrosis was observed at 16 weeks (Supplementary Data, Figure S1). However, changes in LOXL2 were concomitant with echocardiography data. This was confirmed by Alex who further argued that the db/db mouse model in the C57BL6J background is a pathophysiological relevant model to study diabetes-induced cardiac dysfunction as it displays a marked increase in cardiac risk predicters, however, cardiac fibrosis using this specific model are only detected at 6 months only (24 weeks)^24^. Taken together, these results confirm that this model displays a T2DM phenotype, whilst high cholesterol and LDL levels are important risk parameters for CVD development, thus, making it a clinically relevant model to test the identified biomarkers.

During disease progression, cardiac hypertrophy is usually characterized by an increase in interstitial fibrosis with augmented triglycerides and LDL affecting oxidative phosphorylation, myocardial structure as well as gene and protein expression of essential genes within this process^22^. In the present study, we found an increase in mRNA expression of *LOXL2,* after 11 weeks of age, which was concomitant with an impairment in LV diastolic function. This was the first study to show such an increase in diabetic cardiomyopathy. The observed increase at 11 weeks of *LOXL2* expression was confirmed by various other HF studies other than diabetes, where the authors showed an increase in mRNA expression of *LOXL2* was positively correlated with increased tissue fibrosis^14,25,26^. Similarly, in a study performed by Zhao, it was argued that serum LOXL2 levels were positively correlated to the degree of left atrial fibrosis in Atrial fibrillation (AF) patients^15^. Furthermore, findings from Lu and later studies by Karthik argued that ETFβ may be modulated during the development of insulin resistance in pancreatic β cells whereas the upregulation reduced cardiovascular dysfunction ^17,27^. Conversely, Boudina showed that after exposing db/db mice to increased palmitate, protein expression levels of ETFβ were significantly decreased, however no coordinate increase in oxidative phosphorylation was observed^28^. Nonetheless, more recently in a study done by Ruiz-Pinto, it was found that susceptible variants in the ETFβ is a major contributor to anthracycline-mediated oxidative stress and myocardial injury. Inferring that decreased ETFβ expression is linked to myocardial dysfunction^29^. Our study made use of a db/db mice model who confirmed previous findings from Yang^14^ and Lu^17^, using different models showing that differential expression of *LOXL2* and *ETFβ* was concomitant to increased ROS. Inferring that LOXL2 and ETFβ might be implicated in the disease pathophysiology of DCM.

During disease progression, increased gluco- and lipotoxicity affect mitochondrial substrate preference ensuing in the enlargement of the heart muscle and hindering the ability of the myocardium to pump blood through the body effectively. As cardiomyopathy progresses, the heart becomes weaker due to reduced ejection fraction and is less able to pump blood through the body effectively. Although the precise age of onset in heart muscle deterioration in DCM is still incompletely understood, the development of LVD can directly be associated with increased fibrosis and development of LV wall thickness and decreased mitral E:A ratio. Mitral E:A ratio and ejection fraction are currently used as measurements of the hearts' ability to function optimally. In our study, we found that our identified serum markers correlated with cardiac dysfunction when the disease was still in its asymptomatic stage. This implies that the change in the circulatory markers (LOXL2 and ETFβ) are as a result of the diabetic state that induces functional changes prior to the onset of any pathological changes. As such, the observed differential expression of the biomarkers is complicit to the maladaptive structural modifications that follow. According to Fuentes-Antras the maladaptive structural changes underlies the observed changes as shown by echocardiography, confirming that the hyperglycaemic state is the cause of LVD^30^.

Furthermore, this study showed that knockdown of either LOXL2 or ETFβ was able to reduce the expression of *COL1A,* a gene associated with increased fibrosis. Increased interstitial fibrosis, in the stressed heart, triggers the formation of enhanced collagen deposition that stiffens the heart muscle, while impairing diastolic relaxation and filling. Increased collagen deposits are not only concomitant to increased COL1A expression but also reduced ejection fraction and LV remodeling. Our data presented, therefore, infers that collagen formation might be modulated by LOXL2 and ETFβ. Our findings are supported by two independent research groups reporting on the role of LOXL2 and ETFβ and their association with increased fibrosis^14,31,32^. In addition, ROC curve analysis strongly supports that the identified markers were sensitive enough to detect the early onset of DCM, as confirmed by the AUC of 0.813 which is an indication of a good predictive test.

## Study limitations

A limitation to this study was the inability to show the role ETFβ plays in mitochondrial bioenergetics and the effect a loss in ETFβ enzyme activity has on the respiratory chain, and subsequent ATP production. According to Boudina^28^, ETFβ is an electron acceptor that transfers electrons between Acyl-CoA substrates to the main respiratory chain, ubiquinone, which links the oxidation of fatty acids to the mitochondrial respiratory system. As such, a loss of function or decreased ETFβ is known to alter cardiac mitochondrial flux, which consequently is the hallmark of DCM. To address this future studies should consider assessing the link between ETFβ and respiratory chain function and mitochondrial ultrastructural abnormalities within the disease state.

Furthermore, it would have been of interest to follow disease progression from asymptomatic until a late stage of DCM, using LOXL2 and ETFβ as serum biomarkers. Also, scarring as a consequence of hyperglyceamia is a progressive disease that worsens over time, with no cure and attaining treatment early might slow progression whilst maintaining the quality of life of the affected individual. Based on findings presented including studies performed by Yang^14^ and Lu^17^ it could be suggested that those diabetic patients (in this study reference is made to mice) with early dysregulation of serum LOXL2 and ETFβ levels may be treated prophylactically in order to slow the progress of CVD. The proposed implementation strategy might be cost-intensive, however, in time patients might benefit from such treatment through increased life expectancy. As such, a follow-up human study should be initiated to prove such a hypothesis as the scope of this study did not encompass this important aspect. In the case of whether the identified markers can be used as either a preventative or even therapeutic intervention, it requires further investigation in order to support the clinical implication of the attained results.

## Conclusion

It is well known that that increased hyperglycemia and augmented lipid accumulation are the main drivers of myocardial dysfunction and subsequent heart failure. In our study, a change in previously identified possible cardiac markers, LOXL2 and ETFβ, was confirmed in the serum of db/db mice and correlated with increased LVDD, as observed with high-resolution Doppler echocardiography^14,17,27,28^. We observed a significant increase in FBG and serum lipids levels from 9 weeks onwards. This was concomitant with increased LOXL2 and decreased ETFβ serum levels at 9 and 11 weeks, respectively. Confirming a diabetic phenotype that was sustained until termination. As such, it would have been of interest to investigate if the identified changes occur only in the diabetic phenotype, regardless of whether they develop CVD and/or whether only the diabetic animals that eventually develop CVD had differentially regulated levels of the identified markers. Further investigation would need to be conducted to address this issue.

Furthermore, this study brings further insights to suggest that LOXL2 and ETFβ might be implicated in the pathophysiology of DCM. Mechanistically, we propose that both identified biomarkers were able to regulate the expression of COLA1, a known cardiac fibrotic marker^14^. Most importantly, our data strongly suggest that the combined use of LOXL2 and ETFβ displayed a good predictive power to detect the early onset of DCM and as such, support that the disease progression can be slowed through early intervention.

## Materials and methods

### In silico prediction of DCM biomarkers

#### Data acquisition and processing

In order to identify candidate genes differentially expressed in diabetic cardiomyopathy (DCM), the ArrayExpress database (https://www.ebi.ac.uk/arrayexpress) was screened for studies with publicly available microarray datasets belonging to Homo sapiens over 35 years of age. These studies included healthy controls and patients suffering from either type 2 diabetes mellitus (T2DM) or cardiovascular disease (CVD). To provide specificity for the disease state of interest (DCM), studies involving ischemia, CAD and hypertension were excluded; while studies inclusive of LVD and left ventricular ejection fraction (EF) were included with no restriction on ethnic group, gender, tissue or cell type.

For studies, utilising the Affymetrix and Agilent platform, raw datasets were extracted from Gene Expression Omnibus (GEO) using R scripting and the R package, *GEOquery*. Samples within these datasets fulfilling the specified criteria were then Robust-Multi array Average (RMA) normalised using the R package, *Simpleaffy*. Additionally, studies using a custom spotted oligonucleotide array was also included. These raw datasets were imported into R using the *ArrayExpress* package, while the Limma package was used for normalisation. Subsequently, microarray probe identifiers (IDs) in all the processed datasets were converted to Ensemble IDs or gene names, using a custom Python script. Probe sets, mapping multiple Ensembl IDs and technical replicates, were averaged to resolve probe redundancy using R and Ruby scripting.

#### Dataset integration and candidate gene identification

Differential expression analysis was performed in three stages: 1) to obtain differentially expressed genes (DEGs) for T2DM only, 2) to obtain DEGs for CVD only and 3) to obtain DEGs for integrated datasets from both T2DM and CVD, representing the DCM group. To this end, common Ensemble IDs were combined using a quantile discretization (QD) data binning approach, which integrates different microarray data at the gene expression level. In this study, a QD range of 128 was used to combine datasets in the T2DM and CVD subgroups only for statistical differential expression analysis, while a QD range of 1,028 was used for the integration (i.e. DCM) subgroup. Genes with a *p*-value of < 0.05 were regarded as significantly different using the Wilcoxon Rank Sum Test with Bonferroni and Benjamin-Hochberg correction for multiple testing. To prevent bias and due to limited samples, only the top 2 genes based on their Wilcoxon score were used for subsequent biomarker analysis.

### In vivo validation

#### Animals sample size and randomization

Male C57BLKS/J leptin-receptor-deficient mice were obtained from Jackson’s Laboratories (Sacramento, USA) and housed at the Primate Unit and Delft Animal Centre (PUDAC) of the South African Medical Research Council (SAMRC) in a controlled environment with a 12 h light/dark cycle in a temperature range of 23-25ºC (relative humidity: ∼50%). The mice received standard laboratory chow pellets (Afresh Vention, Cape Town, SA) ad libitum and had free access to water. At 6 weeks, based on their genetic background, mice were divided into 2 groups: (1) homozygous leptin receptor-deficient diabetic (db/db, n = 40) mice and (2) heterozygous leptin-receptor-deficient non-diabetic wild type controls (db/+, n = 40). Each group was then further divided into a total of 5 subgroups (week 6, 9, 11, 14, 16), with n = 6–8 animals per subgroup. After each week, (6, 8, 9, 11, 14 and 16) high-resolution Doppler echocardiography was performed, and animals were terminated to collect blood and heart tissue for subsequent serum biomarker analysis. Ethical clearance for the use of animals was obtained from South African Medical Research Council (SAMRC) (ECRA 07/13) and all experiments were conducted in accordance with the National Institute of Health Guide for the Care and use of laboratory animals.

#### Echocardiography analysis

The mice were placed in a holding chamber and gassed with 1.0–2.5% isoflurane, whereafter they were removed and positioned in the supine position on a warming pad. Closed chest high-resolution Doppler echocardiography was performed with a VEVO 2,100 ultrasound system (Fujifilm, Visualsonics, Ontario, Canada) and a 30 MHz linear array transducer at the respective time intervals. Left ventricular internal diameter, interventricular septum thickness, posterior wall thickness were measured in systole and diastole (Figure S2). EF was measured as a marker of systolic function. Mitral E and A wave flow velocity, E:A ratio, deceleration time of mitral E wave velocity and isovolumic relaxation time (IVRT) were assessed as measurements of LVD using Doppler echocardiography (Figure S2). All measurements were made off-line on the mean of at least three consecutive cardiac cycles with the software resident on the ultrasound system.

#### Body weights and fasting blood glucose levels

Body weights were measured weekly, while 4 h FBG levels were monitored by tail prick using a OneTouch Select glucometer (Lifespan, Milpitas, CA, USA).

#### Cardiovascular disease risk predictors

On the day of termination, mice were anaesthetised with halothane before blood was collected from the abdominal vena cava for subsequent lipid profile and serum biomarker analysis. Serum was isolated from peripheral blood by centrifugation at 4,000 g for 15 min. Thereafter, the serum was removed and divided into two parts; one part was sent to PathCare Medical Diagnostic Laboratories (Cape Town, RSA) for total cholesterol, triglycerides and LDL analysis; while the other half was stored at -80ºC for subsequent serum biomarker analysis.

#### Detection of LOXL2, ETFβ and proBNP in serum

LOXL2, ETFβ and NT-proBNP concentrations were measured with enzyme-linked immunosorbent assay (ELISA) (MYbioscource, Berlin, Germany), according to the manufacturer’s instructions.

### Functional analysis using an in vitro model

#### Cell culture

H9c2 cardiomyoblasts were cultured in supplemented Dulbecco’s Modified Eagle’s Medium (10% fetal bovine serum, 100 µg/mL penicillin and 100 µg/mL streptomycin) under standard tissue culture conditions (37 °C in humidified air and 5% CO2). Cells were seeded in a 6-well plate at a seeding density of 2 × 10^4^ cells/well and left for 2–3 days until confluent. On day 4, cells were utilised for transfection experiments using siRNA.

#### Knockdown of LOXL2 and ETFβ using small Interfering RNA (SiRNA)

Knockdown experiments for the two selected candidate biomarkers were done using a Lipofectamine RNAimax reagent (ThermoFisher Sceintific, Inc, Waltham, MA, USA), according to the manufacturer’s instructions. Briefly, H9c2 cells at approximately 70% confluence were transfected with either siLOXL2, siETFβ or scrRNA for LOXL2 or ETFβ for 24 h, respectively. Thereafter, transfected cells were exposed to both high glucose (HG; 33 mM) and palmitate (PAL; 100 µM) for 6 h. The degree of knockdown after 24 h of transfection was confirmed by RT-PCR. Non-transfected cells, exposed to either 5.5 mM glucose or 33 mM glucose, served as controls, respectively.

#### Gene expression analysis using quantitative real-time polymerase chain reaction (qRT-PCR)

Total RNA was extracted using the TRIzol method (ThermoFisher Scientific Inc, Waltham, MA, USA), and subsequently purified with an RNeasy mini kit according to the manufacturer’s instructions. The extracted RNA was treated with DNase1 (Applied Biosystems, Foster City, CA, USA) to eliminate genomic DNA contamination and reversed transcribed to generate cDNA with a Superscript first-strand cDNA synthesis kit (Applied Biosystems, Foster City, CA, USA) as per previously described protocol^33^. Thereafter, qRT-PCR analysis was performed using 5 µL Taqman Universal PCR master mix, 0.5 µL predesigned and optimized TaqMan gene expression assays (*LOXL2*: Mm00804740_m1; *ETFβ*: Mm005033401_m1) and 1 µL cDNA on an ABI 7,500 Instrument (Applied Biosystems, Life Technologies, USA). The PCR reaction was as follows: 50 °C for 1 min and 95 °C for 10 min; followed by 95 °C for 15 s and 60° C for 30 s (40 cycles). Gene expression data was normalized to that of Actin-β (*ACT-β*) and hypoxanthine–guanine phosphoribosyltransferase (*HPRT*) and relative gene expression was calculated using the standard curve method.

### Statistical analysis

All data obtained throughout this study are expressed as the mean ± standard error of the mean (SEM) where appropriate. Results for in vitro experiments were expressed as the mean of three independent biological experiments, with each experiment containing at least three technical replicates, unless otherwise stated. For in vivo experiments, each treatment group included six mice. Differences between the means of groups were first tested for normality with Shapiro–Wilk tests. In the instances where data sets were normally distributed, they were assessed for statistical significance using one-way or two-way multivariate ANOVA and Student t-tests, while non-parametric Mann–Whitney U tests were used to analyse data sets deviating from^1^ a normal distribution. To evaluate the diagnostic potential of each biomarker, principal component (PCI) analysis was performed by combining the data for the identified biomarkers and receiver operating characteristic (ROC) curve analysis, where an area under the curve (AUC) greater than 0.5 indicated diagnostic value. Subsequently, the ROC curve analysis was used to calculate corresponding sensitivity and specificity. To measure the strength and nature of the relationship between the expression levels of the candidate biomarkers and mitral E:A ratio, correlation analysis was performed using a Pearson and Spearman coefficient test. Statistical analyses were performed using GraphPad Prism software version 5.00 (GraphPad Software, Inc., La Jolla, CA, USA). In all instances, statistical significance was inferred for a *p*-value less than, or equal to 0.05.

## Supplementary information

Supplementary Information.
