# Supplementary material for: Identification of potential biomarkers for predicting the early onset of diabetic cardiomyopathy in a mouse model
Source: Sci Rep. 2020 Jul 23;10:12352. doi: 10.1038/s41598-020-69254-x (PMC7378836; doi:10.1038/s41598-020-69254-x)
Supplement: Supplementary file 1 — Supplementary Information. [file 41598_2020_69254_MOESM1_ESM.docx]

IDENTIFICATION OF POTENTIAL BIOMARKERS FOR PREDICTING THE EARLY ONSET OF DIABETIC CARDIOMYOPATHY IN A MOUSE MODEL

Rabia Johnson^1,2*^, Xolisa Nxele^1,3^, Martin Cour^4,5^, Nonhlakanipho Sangweni^1,2^, Tracey Jooste^1,2^, Nkanyinso Hadebe^5,6^, Ebrahim Samodien^1^, Mongi Benjeddou^3^, Mikateko Mazino^7^, Johan Louw^1,8^ and Sandrine Lecour^5^,

^1^Biomedical Research and Innovation Platform (BRIP), South African Medical Research Council (SAMRC), Tygerberg 7505, South Africa.

^2^Division of Medical Physiology, Faculty of Health Sciences, Stellenbosch University, Tygerberg 7505, South Africa.

^3^Department of Biotechnology, University of Western Cape, Department of Medical Physiology, Stellenbosch University, Tygerberg 7507, South Africa.

^4^Hospices Civils de Lyon, Hôpital Edouard Herriot, Service de Médecine Intensive-Réanimation, Lyon Cedex 03, France

^5^Hatter Institute for Cardiovascular Research in Africa (HICRA), University of Cape Town, Observatory, South Africa.

^6^Department of Anaesthesia, University of Cape Town, Observatory, South Africa.

^7^Biostatistics Research Unit, South African Medical Research Council (SAMRC).

^8^Department of Biochemistry and Microbiology, University of Zululand, KwaDlangezwa 3886, South Africa.

* Correspondence should be addressed to rabia.johnson@mrc.ac.za

**SUPPLEMENTARY MATERIALS**

**Materials and Methods**

**Immunohistochemistry of LOXL2 and Masson’s Trichrome staining**

Heart tissues were weighed and fixed in phosphate-buffer saline with 4% paraformaldehyde (Merck-Millipore, Billerica, USA) for a minimum of 16 hrs, prior to dehydration and paraffin embedding using a Leica TP 1020 automated processor (Leica Biosystems, Buffalo Grove, USA). Following this, 4 µm thick sections were cut and stained with Hematoxylin and Eosin (H&E) as well as Masson's trichrome staining as per manufacturer’s instructions (Sigma, St. Louis, MO, USA). Additionally, sections were also probed with primary rabbit antibodies for LOXL2 (1:200, Abcam) followed by staining with a secondary anti-rabbit (Sigma, St. Louis, MO, USA) antibody according to manufacturer’s instructions.

**Results:**

Immunostaining of embedded tissue section with H&E showed no visible signs of myocardial tissue damage at 16 weeks. However, myocardial tissue staining with anti-LOXL2 showed increased protein expression at 16 weeks (Figure S1). In order to correlate increased circulating LOXL2 expression with fibrosis, tissue section was stained with Masson’s Trichrome at 16 weeks. Results obtained showed a moderate increased in cardiac fibrosis at 16 weeks only (Figure S1), with no signs of fibrosis detected at 6-11 weeks (data not shown).

**SUPPLEMENTARY FIGURES**

**Obese (db/db**) **16-week-old mice**

**Wild type (db/+) 16-week-old mice**


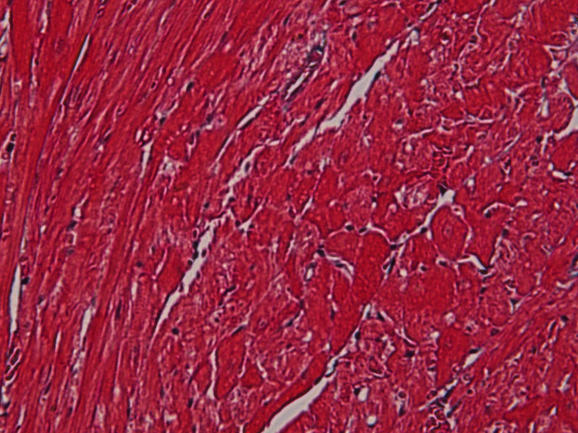

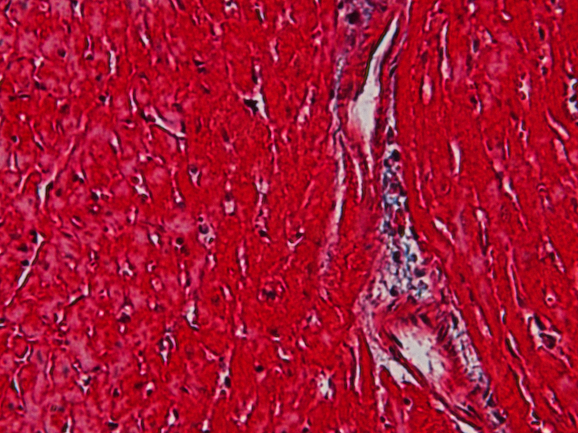


H&E Stain


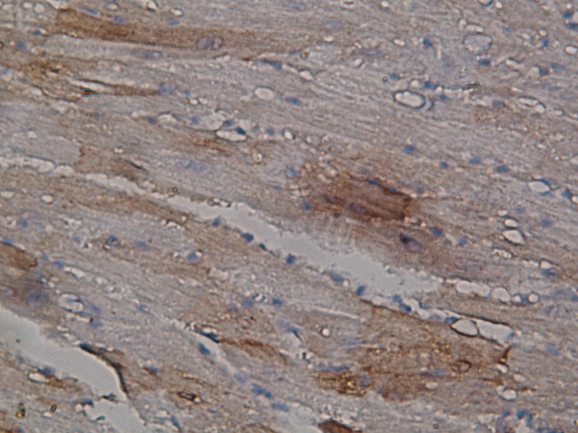

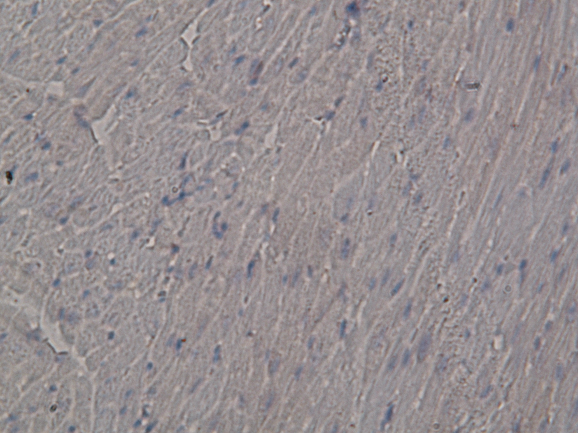


LOXL2


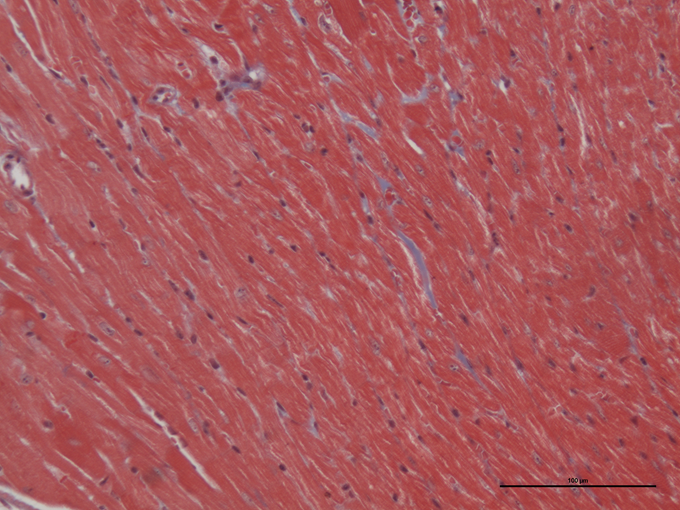

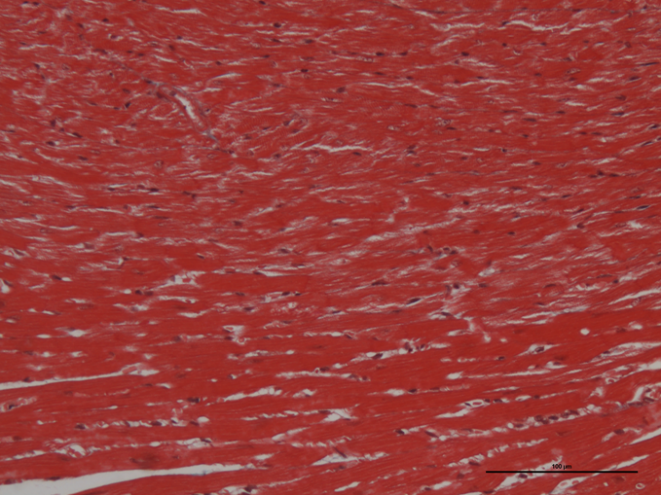


Masson

Trichrome stain

**Figure S1:** **LOXL2 expression correlates with a moderate fibrosis in db/db mice heart.** Representative immunostaining of cardiac tissues with Hematoxylin and Eosin (H&E), anti-LOXL2 (brown) and Masson's trichrome (blue) staining. LOXL2 in in the interstitial space stain brown, whilst collagen accumulation is shown in blue (black arrow). The myocardial architecture images were magnified ×400.


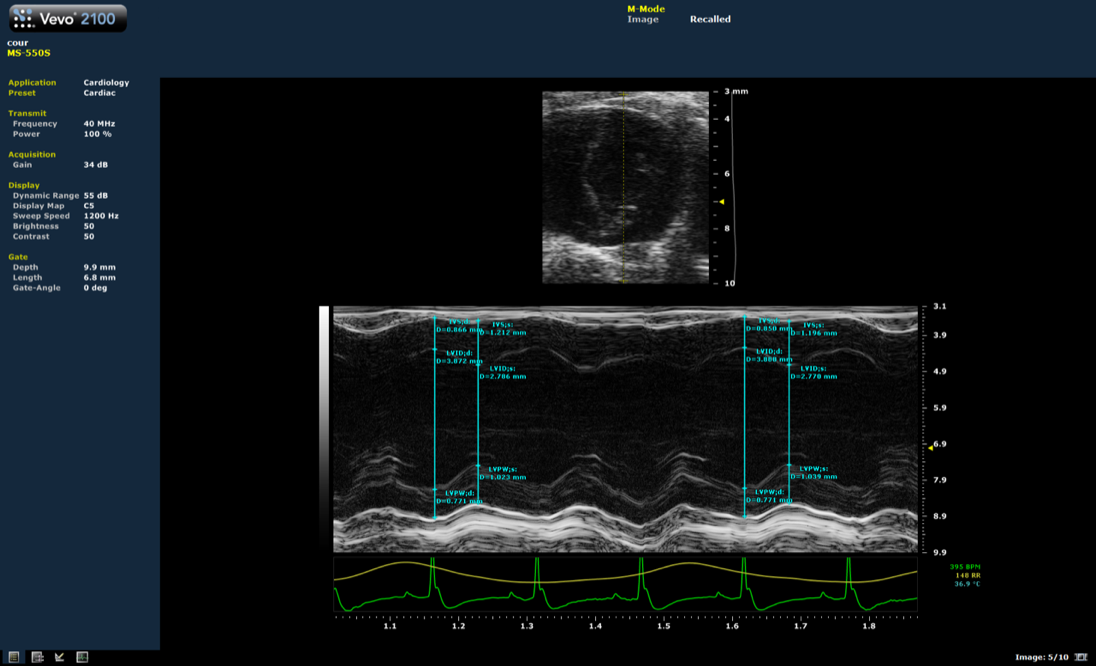


A.

B.


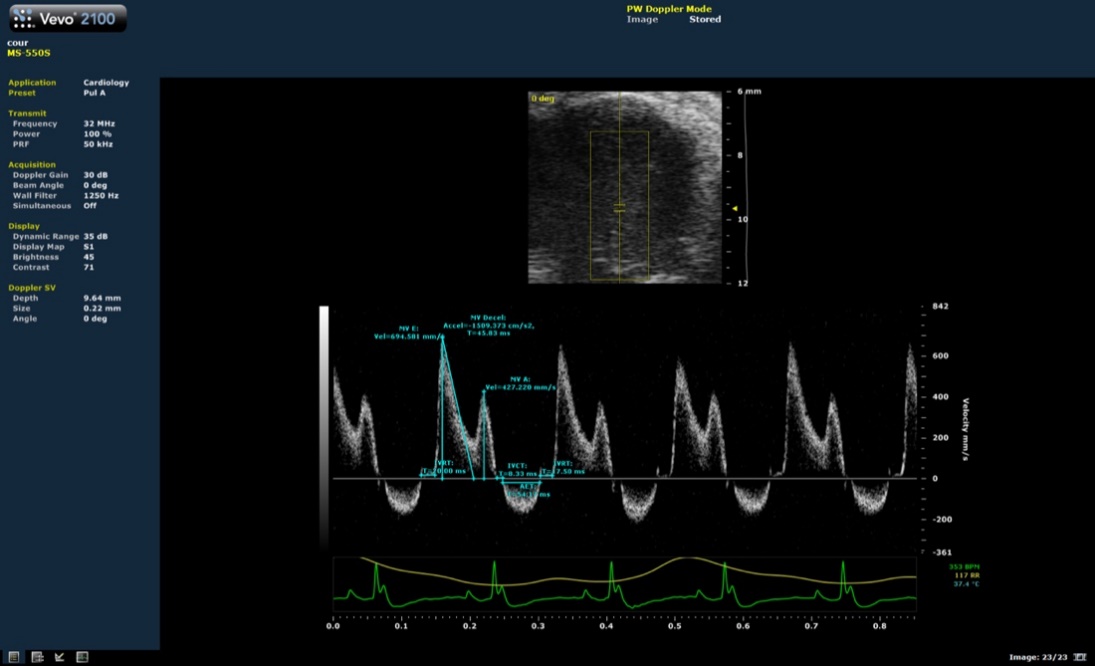


**Figure S2:** **Representative images for measurements of left ventricle dimensions (M-mode) and for measurements of mitral flow parameters (Doppler).**

Interventricular septum thickness (IVS), left ventricle (LV) internal diameter (LVID), left ventricle posterior wall thickeness (LVPW) were obtained from the parasternal short axis view at the level of the papillary muscle using M-mode (A). Peak E and A flow velocity of mitral inflow, E deceleration time (MV decal) and isovolumic relaxation time (IVRT, time from the end of the aortic outflow and mitral inflow) were measured using Doppler echocardiography (B).

**SUPPLEMENTARY TABLES**

**Supplementary Table 1 - High resolution echocardiography**

|  | **6 weeks** | | **9 weeks** | | **11 weeks** | | **14 weeks** | | **16 weeks** | |
| --- | --- | --- | --- | --- | --- | --- | --- | --- | --- | --- |
|  | **Wild type** (db/+)  (n=8) | **Obese** (db/db)  (n=8) | **Wild type** (db/+)  (n=7) | **Obese** (db/db)  (n=8) | **Wild type** (db/+)  (n=8) | **Obese** (db/db)  (n=8) | **Wild type** (db/+)  (n=6) | **Obese** (db/db)  (n=6) | **Wild type** (db/+)  (n=8) | **Obese** (db/db)  (n=8) |
| **Heart Rate** | 408±52 | 397±17 | 408±43 | 372±26 | 380±35 | 388±36 | 383±34 | 353±20 | 410±35 | 398±28 |
| **Left ventricle** |  |  |  |  |  |  |  |  |  |  |
| IVS, d (mm) | 0.71±0.06 | 0.72±0.06 | 0.78±0.11 | 0.75±0.07 | 0.81±0.14 | 0.79±0.07 | 0.83±0.07 | 0.75±0.07 | 0.82±0.08 | 0.77±0.09 |
| IVS, s (mm) | 1.15±0.07 | 1.22±0.15 | 1.23±0.32 | 1.17±0.10 | 1.28±0.32 | 1.15±0.05 | 1.27±0.13 | 1.07±0.11 | 1.36±0.20 | 1.08±0.11 |
| LVID, d (mm) | 3.68±0.29 | 3.79±0.24 | 3.81±0.32 | 3.75±0.18 | 4.04±0.25 | 3.91±0.32 | 3.99±0.26 | 4.05±0.27 | 4.16±0.22 | 3.95±0.42 |
| LVID, s (mm) | 2.29±0.30 | 2.23±0.24 | 2.47±0.47 | 2.49±0.22 | 2.56±0.46 | 2.71±0.25 | 2.96±0.37 | 3.07±0.22 | 2.67±0.26 | 2.92±0.29 |
| LVPW, d (mm) | 0.68±0.07 | 0.77±0.17 | 0.73±0.05 | 0.70±0.13 | 0.78±0.13 | 0.81±0.05 | 0.78±0.09 | 0.80±0.07 | 0.73±0.09 | 0.79±1.06 |
| LVPW, s (mm) | 1.06±0.15 | 1.12±0.18 | 1.08±0,17 | 1.05±0.19 | 1.16±0,24 | 1.12±0.11 | 1.14±0.12 | 1.01±0.07 | 1.14±0.15 | 1.06±0.16 |
| LV mass (mg) | 78±11 | 90±6 | 99±10 | 94±21 | 119±17 | 113±11 | 120±15 | 115±11 | 121±21 | 113±30 |
| **Diastolic function** |  |  |  |  |  |  |  |  |  |  |
| E wave (mm/s) | 808±116 | 734±61 | 822±108 | 652±85 | 792±106 | 694±61* | 710±82 | 582±101* | 775±66 | 606±154** |
| A wave (mm/s) | 477±110 | 437±48 | 437±133 | 415±78 | 421±124 | 421±41 | 388±68 | 397±59 | 398±76 | 441±92 |
| IVRT (ms) | 13±3 | 13±7 | 11±1 | 15±5 | 13±3 | 17±3 | 17±3 | 27±6** | 15±2 | 21±6** |
| E_DT_ (ms) | 22±9 | 27±6 | 22±7 | 26±6 | 21±10 | 24±8 | 25±3 | 36±7* | 21±6 | 32±4** |

IVS: interventricular septum; d: diastole; s: systole; LVID: left ventricle internal diameter; LVPW: left ventricle posterior wall; LV mass: left ventricle mass; IVRT: isovolumetric relaxation time; E_DT_: E wave deceleration time. Data are expressed as mean±SD. ^*^p < 0.05, ^**^p < 0.01 compared to aged matched wild type control using Mann-Whitney test.
